# Supplementary material for: Comparison of Four Assays That Measure Antibodies to Ebola Virus Glycoprotein
Source: bioRxiv. 2026 Mar 20:2026.03.18.708022. Preprint. [Version 1] doi: 10.64898/2026.03.18.708022 (PMC13015300; doi:10.64898/2026.03.18.708022)
Supplement: Supplement 1 [file media-1.pdf]

1. ASSAY COMPARISONS

MATERIALS AND METHODS

nFANG

Anti-EBOV $GP_{1,2}$ IgG titers on all 298 samples were measured at IRF-Frederick using the FANG ELISA following the NFANG protocol, as previously described (1) (2). In detail, microtiter plates (Thermo Fisher, X1530419 NT521019) were coated with the recombinant Ebola glycoprotein and allowed to incubate at 4 °C for at least 14 hours in the absence of light. The starting dilution of 1:62.5 was prepared using an ELISA Diluent (ED) containing 5% weight-by-volume dry milk (LabScientific, Highlands, NJ, USA) and 0.1% volume-by-volume Tween-20 (FANG wash buffer) (Sigma Aldrich, St. Louis, MO, USA) in phosphate buffered saline (Gibco, Gaithersburg, MD, USA). The ED was used to prepare the reference standard (calibrator) in a dilution block as an 11-point, 1:2 dilution series. In a different dilution box, 5 µL of human serum samples were diluted in 495 µL of ED in duplicates, and an 8-point dilution series was performed. The same dilution series was used for high and low concentration quality controls (Battelle), and pooled naïve human serum (Sera Care, Milford, Ma) was used as a negative control. Thawed EBOV GP-coated plates were washed three times with 300 µL phosphate buffered saline with 0.1% volume-by-volume Tween-20, and 100 µL per well of the serially diluted reference standard, samples (sample replicates plated on different plates), and controls were added and incubated for one hour in 37 °C with absence of light. The plates were subsequently washed three times with FANG wash buffer followed by addition of 100 µL per well of the horseradish peroxidase-conjugated anti-human IgG secondary antibody (Thermo Fisher Scientific, Walkersville, MD, USA). Plates were incubated for another hour at 37 °C in absence of light before washing again three times with 300 µL of FANG wash buffer and adding TMB substrate (Thermo Fisher Scientific, Walkersville, MD, USA). Following a 30-minute incubation at room temperature in the absence of light, 100 µL of stop solution - sulphuric acid (Thermo Fisher Scientific, Walkersville, MD, USA) was added. Plates were measured in a SpectraMax Plus 384 colorimeter plate reader (Molecular Devices, Sunnyvale, CA, USA) at 450 nm. Individual well background optical densities were measured at 650 nm and subtracted from the 450 nm optical density using the Softmax 7.1 version software (Molecular Devices, Sunnyvale, CA, USA).

Q2

In addition, anti-EBOV $GP_{1,2}$ IgG titers were measured on the same 298 samples using the newly developed Ebola GP Simoa Planar assay on an SP-X Automated Immunoassay Analyzer (Quanterix). For the Simoa assay, kits were removed from the refrigerator and brought to room temperature at least 30 minutes before utilization. The 1X wash buffer provided in the kit was used to wash the 96-well plates pre-coated with recombinant Ebola $GP_{1,2}$ (at a titer of 400 µg/mL) on the Simoa™ Microplate Washer. A calibrator standard (provided in the kit) at a seven-point 1:3 serial dilution was created, and high concentration and low concentration quality controls (QCs - provided in the kit) were diluted with sample diluent (provided in the kit) at a 1:4000. Study samples were diluted with the kit-provided sample diluent and measured at serial dilutions of 1:50 and 1:4000. The calibrator, QCs, and samples were added to the plates in duplicates followed by an initial two-hour incubation

with a hydrolyzed microcline lid on the Simoa™ Microplate Shaker at 23 °C and an orbital rotation of 525 RPMs. After incubation, the plates were washed, and a biotinylated antibody was added followed by a 30-minute incubation on the Simoa™ Microplate Shaker as described above. After a second plate wash, the Streptavidin-HRP antibody was added to the plates followed by a 30-minute incubation on the Simoa™ Microplate Shaker and a final plate wash. Super Signal® Substrate was made using 3mL of super signal substrate and 3mL Luminol Enhancer , and 50 µL of Super Signal® Substrate was added to each well. The glass bottom of each plate was wiped with a lint-free cloth and the plates were read within four minutes of adding the Super Signal® Substrate using the imager. Captured images were transferred to a USB drive and uploaded to the SP-X Analysis application.

Luminex Kitwik GP Assay

An aliquot of all 298 samples was shipped to IRD Montpellier TransVIHMI (France) to detect anti-EBOV $GP_{1,2}$ IgG antibodies using a Luminex-based protocol described previously (Ayouba et al, JCM, 2017) that was updated with an additional $GP_{1,2}$ antigen, GPkikwit95, used in certain vaccines. To that end, we coupled recombinant EBOV proteins (NP, VP40, GP kikwit95 and GP Mayinga 76) to carboxy-functionalized beads at a ratio of 2µg of recombinant proteins per million of beads. Before use, recombinant protein-coupled beads were vortexed for 30 seconds and diluted to 2,000 beads/µl of assay buffer (phosphate buffered saline (PBS)) containing 0.75 mol/L NaCl, 1% (wt/vol) bovine serum albumin (Sigma Aldrich, Saint-Quentin Fallavier, France), 5% (vol/vol) heat-inactivated fetal bovine serum (Gibco-Invitrogen, Cergy Pontoise, France), and 0.2% (vol/vol) Tween-20 (Sigma-Aldrich)). Tests were performed in 96-well flat-bottom chimney plates (Greiner bio one, Frickenhausen, Germany) and 50 µl of bead mixture was added to each well. The supernatant was aspirated using an automatic plate washer (BioTek 405TS Microplate washer). Next, the wells were incubated with 100 µL of plasma (diluted 1/200 in assay buffer) for 16 hours at 4˚C in the dark on a plate shaker at 400 rpm/min. After five washings with 100 µL of assay buffer, 50 µLl of biotin-labeled anti-human IgG was added (BD-Pharmingen, Le Pont De Claix, France) at a concentration of 4 µg/mL in each well and incubated for 30 minutes in the dark with continuous shaking at 400 rpm/min. Plates were washed five times as above, and 50 µL of streptavidin-R-phycoerythrin (Fisher Scientific/Life Technologies, Illkirch, France) at 4 µg/ml was added per well and incubated for 10 minutes while shaking at 400 rpm/min. After an additional five washes, 150 µL of reading buffer (PBS 1X, 1% de BSA) was added per well to read antigen-antibody reactions on Intelliflex (Luminex Corp., Austin, TX, USA). At least 100 events were read for each bead set, and the results were expressed as median fluorescence intensity (MFI) per 100 beads.

Limits of Detection and Quantitation

Each assay has its specific limit of detection (LOD), lower limit of quantitation (LLOQ), and upper limit of quantitation (ULOQ), but they are not comparable due to different units and the impact of these limits in any sample analyses is very much dependent on the handling of dilutions. We provide them below for accessibility (Table S1.5) but comparing limits across assays is problematic.

TABLES AND FIGURES

Table S1.1. Participant demographics.

Characteristic	PREVAIL 3 contacts N = 29[1]	PREVAIL 3 survivors N = 120[1]	PREVAIL 1 placebo arm N = 50[1]	PREVAIL 1 vaccine arm N = 99[1]
Female, n (%)	17 / 29 (59%)	66 / 120 (55%)	15 / 50 (30%)	28 / 99 (28%)
Age, mean (SD)	28 (15)	34 (14)	34 (13)	34 (11)

Table S1.2. Three participants with high values in all four assays. All participants were from the placebo arm of PREVAIL 1.

Female	Age	nFANG GP	oFANG GP	Q2 GP	Luminex GP Kikwit/ 9510621	Luminex GP Yambukua/ Mayinga	Luminex NP	Luminex VP40
0	22	4.163	4.073	3.568	3.296	3.416	3.961	3.882
0	19	2.965	4.180	4.055	3.522	3.687	3.957	3.365
1	21	4.416	4.560	4.028	3.486	3.554	3.799	4.004

Table S1.3. Deming regression estimates of slope and intercept using different methods for handling readouts that are below the limit of detection. 95% CIs are from 1000 bootstrap samples.

**y**	**x**	**method**	**intercept**	**Intercept 95%CI**	**slope**	**Slope 95%CI**
Q2	oFANG	LOD/2	-0.871	(-0.96, -0.77)	1.150	(1.12, 1.17)
Q2	oFANG	raw values	-0.980	(-1.00, -0.81)	1.181	(1.13, 1.18)
Q2	oFANG	removing below LOD	-0.955	(-0.99, -0.76)	1.174	(1.12, 1.18)
Luminex	oFANG	LOD/2	-0.920	(-1.07, -0.76)	1.251	(1.22, 1.27)
Luminex	oFANG	raw values	-0.972	(-1.11, -0.81)	1.267	(1.23, 1.29)
Luminex	oFANG	removing below LOD	-1.007	(-1.14, -0.82)	1.275	(1.23, 1.30)
Luminex	oFANG	removing above ULOQ	-0.983	(-1.16, -0.84)	1.280	(1.24, 1.32)
Luminex	Q2	LOD/2	0.042	(-0.12, 0.12)	1.083	(1.05, 1.12)
Luminex	Q2	raw values	0.098	(-0.12, 0.12)	1.066	(1.05, 1.12)
Luminex	Q2	removing below LOD	0.098	(-0.12, 0.12)	1.066	(1.05, 1.12)
Luminex	Q2	removing above ULOQ	0.125	(-0.10, 0.15)	1.047	(1.03, 1.12)
nFANG	oFANG	LOD/2	-0.544	(-0.60, -0.40)	1.154	(1.12, 1.17)
nFANG	oFANG	raw values	-0.530	(-0.54, -0.36)	1.153	(1.11, 1.16)
nFANG	oFANG	removing below LOD	-0.158	(-0.30, -0.10)	1.057	(1.03, 1.09)
Q2	nFANG	LOD/2	-0.316	(-0.41, -0.17)	0.993	(0.96, 1.02)
Q2	nFANG	raw values	-0.385	(-0.48, -0.23)	1.010	(0.98, 1.04)
Q2	nFANG	removing below LOD	-0.557	(-0.61, -0.30)	1.053	(0.99, 1.07)
Luminex	nFANG	LOD/2	-0.323	(-0.53, -0.18)	1.083	(1.05, 1.13)
Luminex	nFANG	raw values	-0.344	(-0.61, -0.25)	1.085	(1.06, 1.15)
Luminex	nFANG	removing below LOD	-0.647	(-0.93, -0.42)	1.159	(1.11, 1.23)
Luminex	nFANG	removing above ULOQ	-0.288	(-0.49, -0.20)	1.068	(1.05, 1.11)

Table S1.4. Q2 assay repeat and sample failure rates per study.

**Type**	**PREVAIL 1**	**PREVAIL 3**
Repeat rate: Out of assay range	0.0%	3.4%
Repeat rate: CV	6.0%	3.4%
Repeat rate: QC	10.0%	9.4%
Repeat rate: Total	16.0%	16.2%
Failure rate	0.7%	0.7%

Table S1.5. Assay Limits in EU/ml. Not adjusted for dilutions.

	oFANG	nFANG	Q2	Luminex
LOD	31.74	27.14	0.0009	6.68
LLOQ	55.34	66.96	0.0049	2.99
ULOQ	2,511.89	55,526.77	20	1840.60

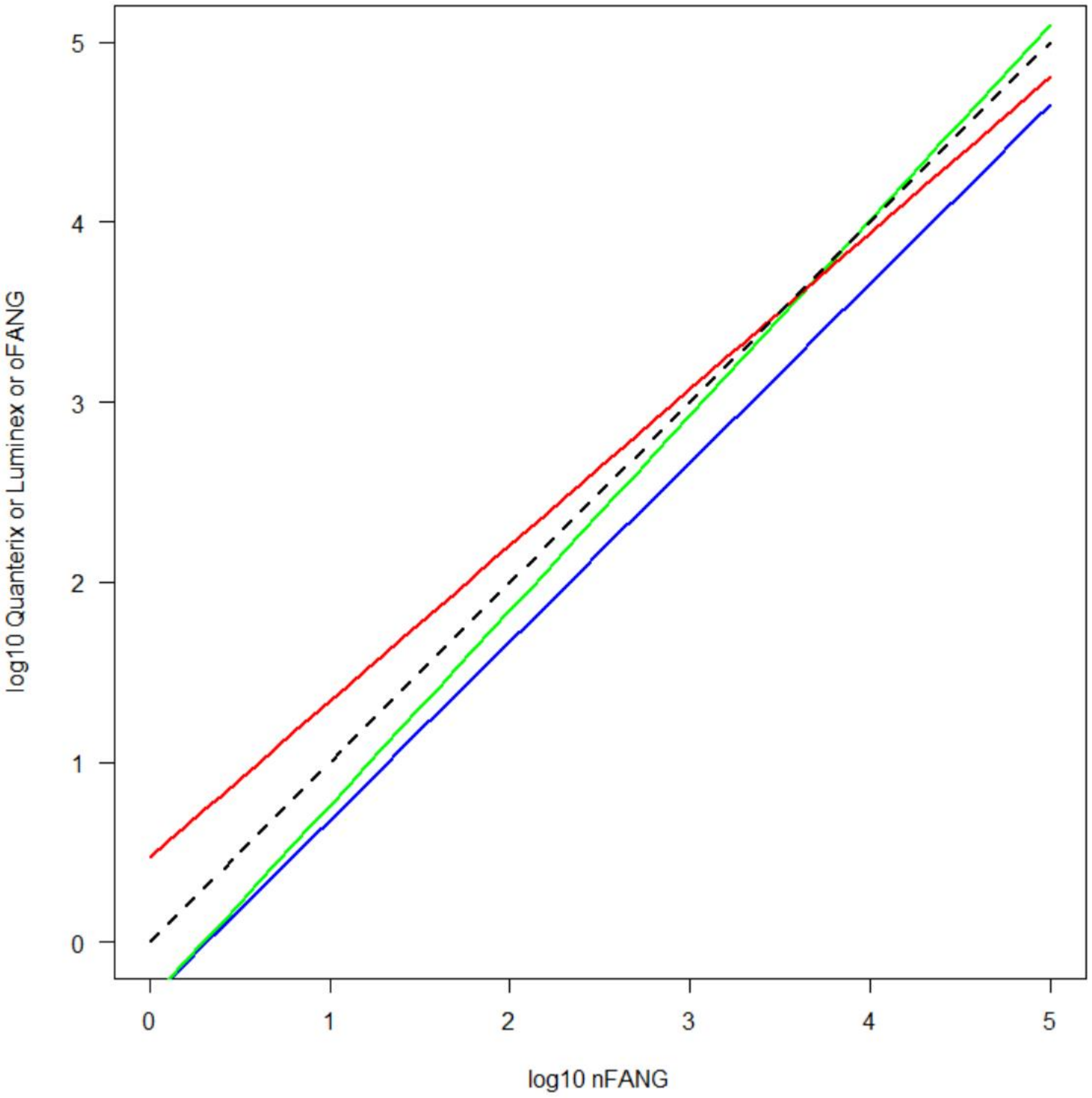

Figure S1.1. Deming regressions of oFANG (red), Luminex (green), and Q2 (blue) assays versus the nFANG assay. The black dashed line is a 45-degree reference line.

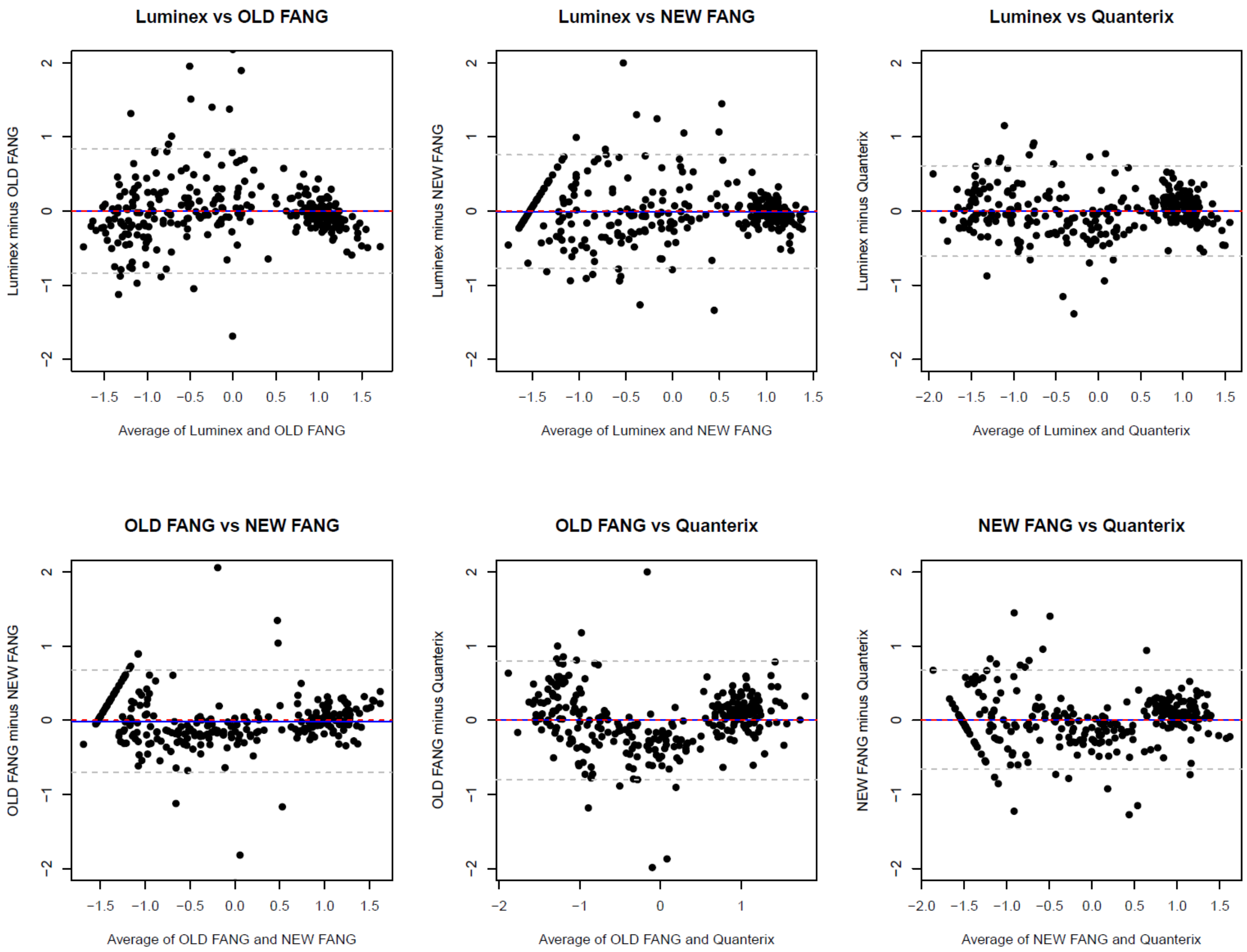

Figure S1.2. Bland-Altman plots for the four different assays (two-by-two comparisons).

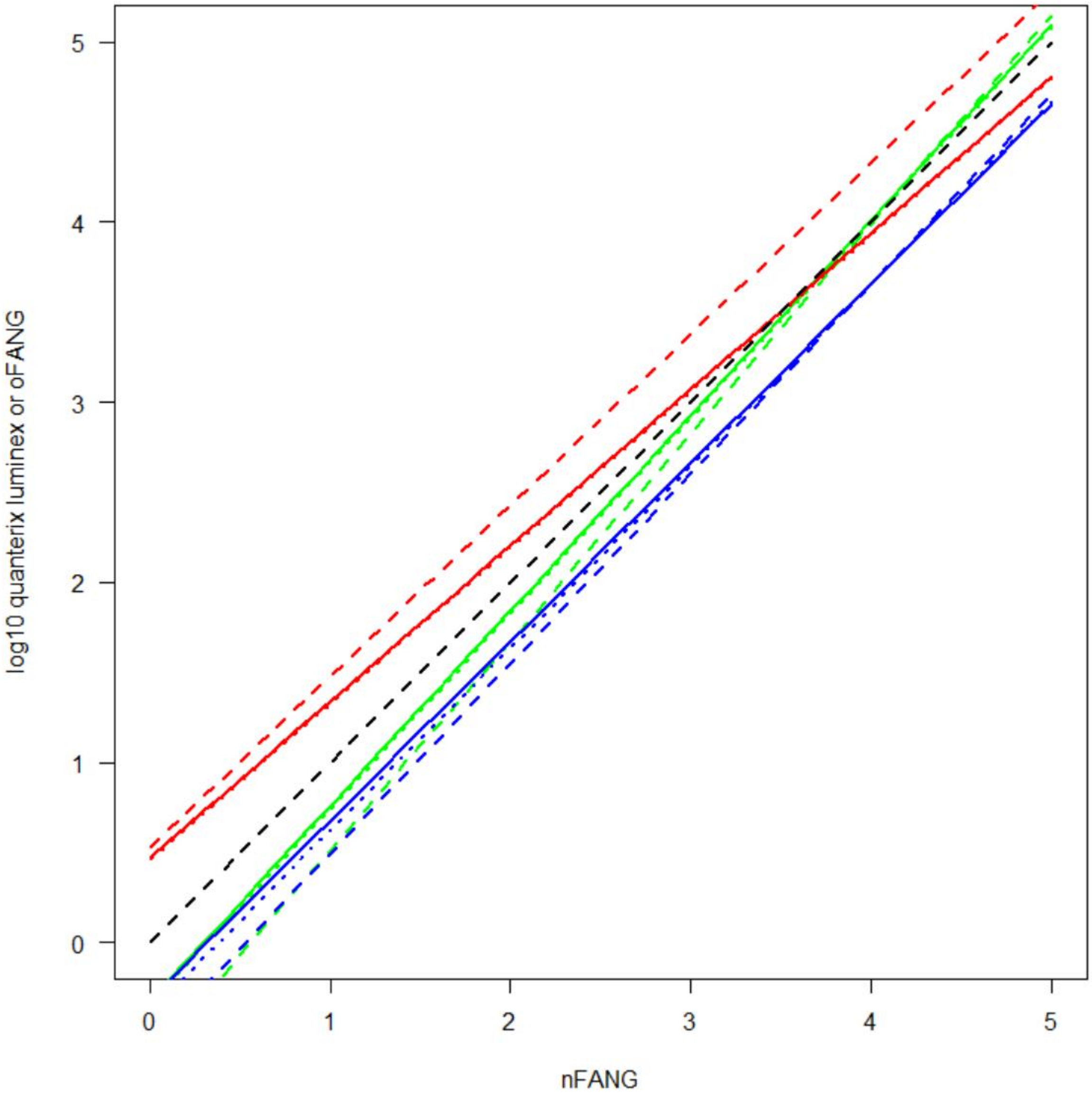

Figure S1.3. Visualization of the conversion equations from nFANG to the other assays: oFANG (red), Luminex (green), and Quanterix (blue) assays versus the nFANG assay under different methods of handling values below LOD (solid=LOD/2, dashed=remove, dotted=raw).

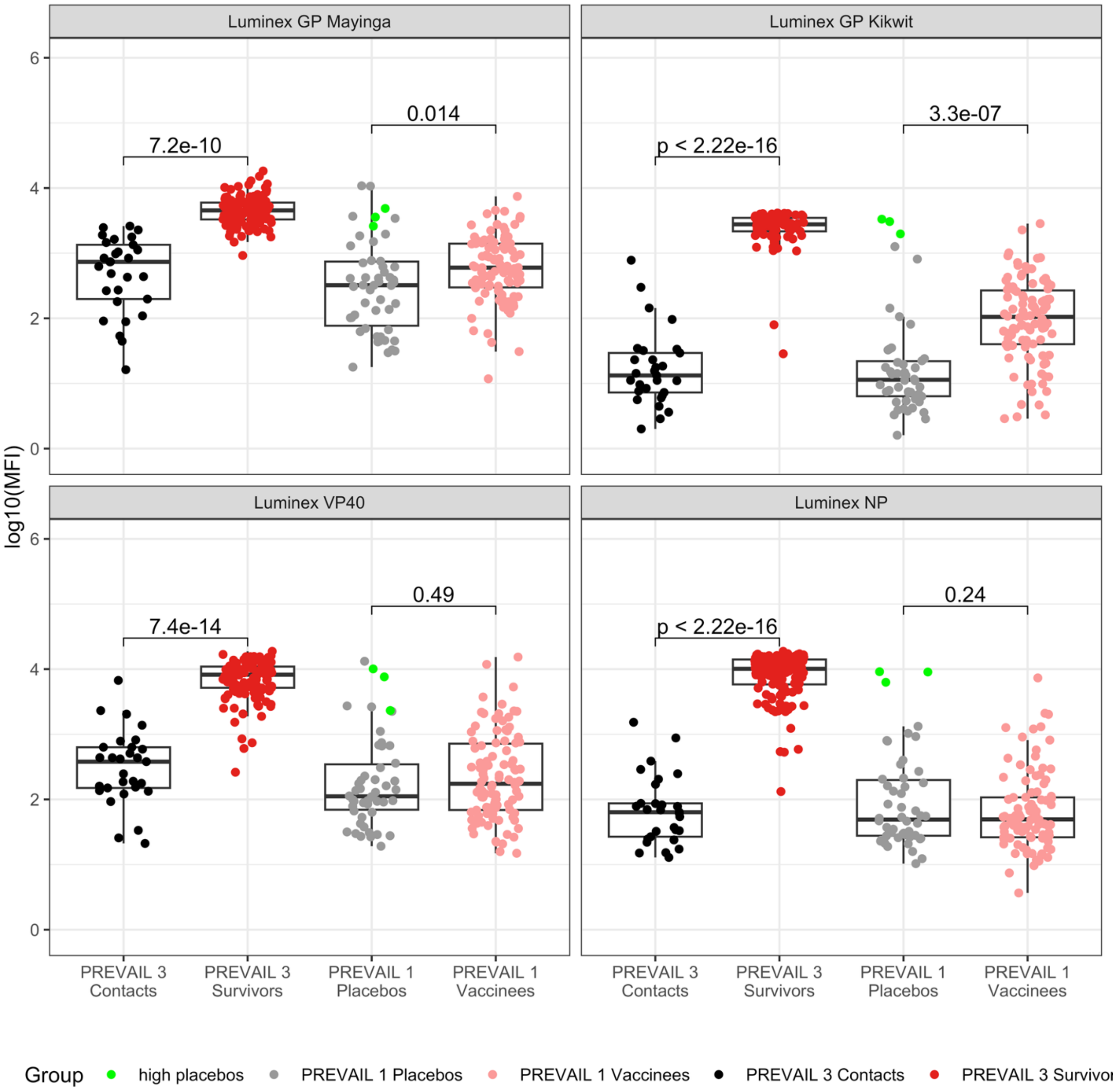

Figure S1.4. Dot plots of the four different targets of the Luminex assay for the four groups of participants; PREVAIL 3 contacts and survivors and PREVAIL 1 vaccine and placebo arms. Three placebo arm participants that read high are denoted by green dots.

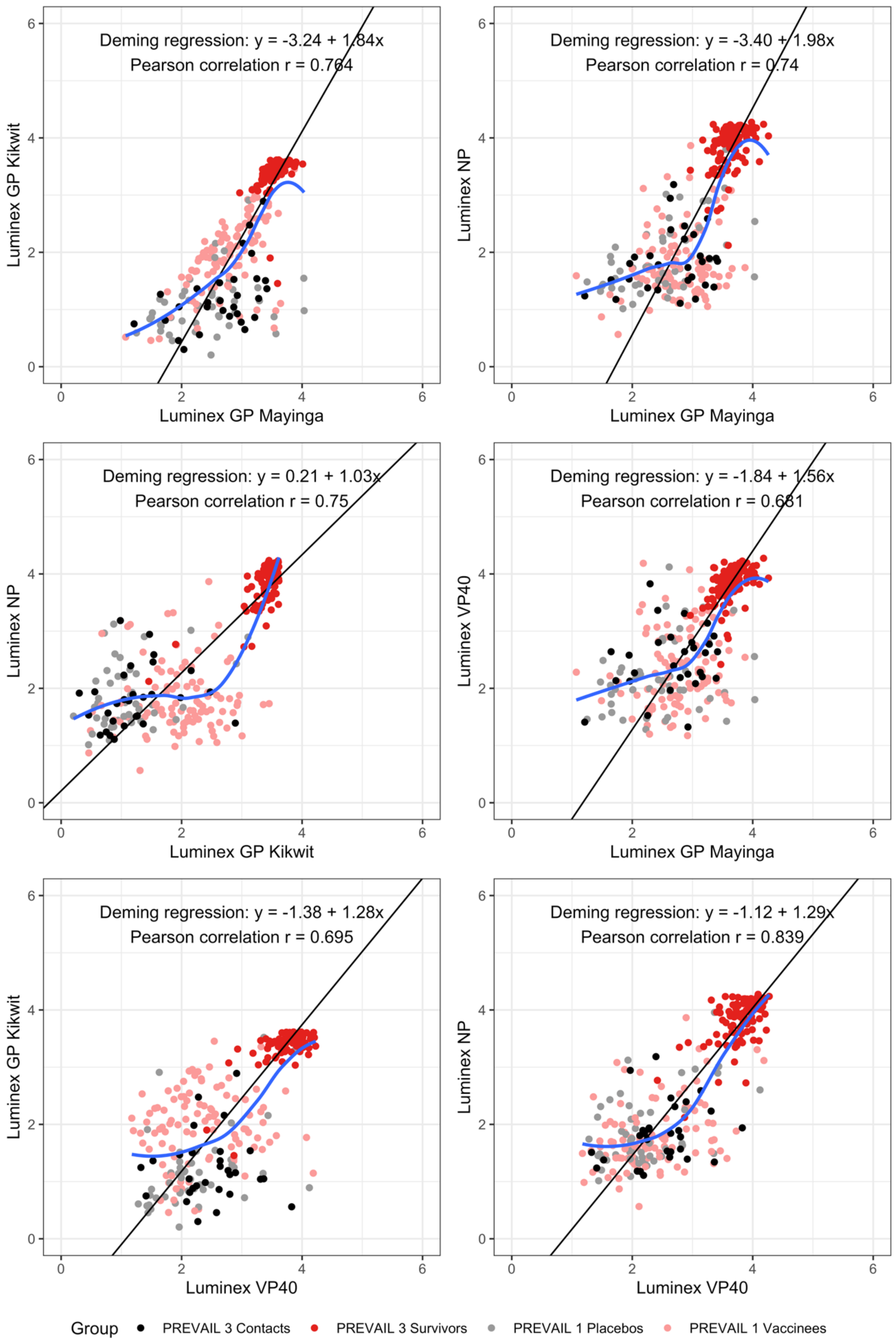

Figure S1.5. Pairwise scatterplots of the four different targets of the Luminex assay.

2. ASSAY VALIDATION: LUMINEX

Materials and methods

Panels sample and controls

We used a panel of 85 human samples of known Ebola virus serostatus to validate our Luminex based multiplex immunoassay. All the human sample used in this work were anonymized with no way to link back to the 85 patients. The panel included 42 sera from the Virology department of the University Hospital of Montpellier were used as negative control and 43 sera coming from a cohort of Ebola virus disease survivor of 2013-2016 outbreak in Guinea were used as positive controls (Ayouba et al. JCM, 2017).

Three pooled serum control with anti-EBOV antibodies concentration information in ELISA Units by milliliter tested by ELISA Fang (Rudge et al, PlosOne, 2019) and two WHO standards (NIBSC) with anti-EBOV antibodies concentration information in international system unit were also used in this study (https://nibsc.org/products/brm_product_catalogue/detail_page.aspx?catid=15/220). To evaluate the assay robustness, we assessed intra- and inter-assay variabilities by testing known samples multiple times, previously tested on the FANG assay as Reference Standard, High Quality Control (HQC) and Low Quality Control (LQC) (Rudge et al. PlosOne, 2019).

Finally, two commercial monoclonal antibodies were used to validate our test: an anti-EBOV GP monoclonal antibody (LA63_CATB-B1092, CD, Shirley NY, USA) and an anti-Ebola surface glycoprotein (KZ52_Ab00960, Absolute Antibody, Wilton, UK).

Luminex based multiplex immunoassay

Recombinant proteins

We used as targets for our Luminex based multiplex immunoassay four commercially available recombinant proteins from different genomic region of orthoebolaviruses (nucleoprotein variant/isolate Makona-Kissidougo-C15 2014 (NP) (40443-V07E1 – Sinobiological (China)); viral protein 40 Makona-Kissidougo-C152014 (VP40) (40446-V07E – Sinobiological (China)); glycoprotein isolate Mayinga 1976 (GP-m) (40304-V08B1– Sinobiological (China)); and glycoprotein strain Kikwit 1995 (GP-k) (EBOVKW95-ENV – The Native Antigen Company, Oxford, UK). The proteins were purchased as lyophilized powders with purity above 90% as defined by SDS-PAGE. They were resuspended in solution and stored as per manufacturer's instruction until use.

Proteins coupling to Luminex Beads

We used our previously described protocol for coupling recombinant proteins to Luminex beads (Ayouba et al. JCM, 2017). Briefly, recombinant proteins (2µg/10^6 beads) were covalently coupled on carboxyl functionalized fluorescent magnetic beads (Luminex Corp., Austin, TX) with the BioPlex amine coupling kit (Bio-Rad Laboratories, Marnes-la-Coquette, France) according to the manufacturer's instructions. We blocked unreacted sites with blocking buffer from the amine coupling kit. Protein-coupled microsphere preparations were washed with PBS and stored in storage buffer from the amine coupling kit at 4˚C in the dark until use.

Luminex based multiplex immunoassay for IgG antibody in serum

Before use, recombinant protein-coupled beads are vortexed for 30 seconds and diluted to 2,000 beads/µL of assay buffer (Phosphate Buffered Saline (PBS) containing 0.75 mol/L NaCl, 1% (wt/vol) bovine serum albumin (Sigma Aldrich, Saint-Quentin Fallavier, France), 5% (vol/vol) heat-inactivated fetal bovine serum (Gibco-Invitrogen, Cergy Pontoise, France), and 0.2% (vol/vol) Tween-20 (Sigma-Aldrich). Tests are performed in 96-well flat-bottom chimney plates (Greiner bio one, Frickenhausen, Germany) and 50 µL of bead mixture is added to each well. Then, the supernatant

is aspirated using an automatic plate washer (BioTek 405TS Microplate washer). Next, wells are incubated with 100 µL of plasma (diluted 1/200 in assay buffer) for 16 hours at 4˚C in the dark on a plate shaker at 400 rpm/min.

After five washings with 100 µL of assay buffer, 50 µL of biotin-labeled anti-human IgG is added (BD-Pharmingen, Le Pont De Claix, France) at a concentration of 4 µg/mL in each well and incubated for 30 minutes in the dark with continuous shaking at 400 rpm/min. Plates are washed five times as above, and 50 µL of streptavidin-R-phycoerythrin (Fisher Scientific/Life Technologies, Illkirch, France) at 4 µg/ml are added per well and incubated for 10 minutes while shaking at 400 rpm/min. The washer performs another five washes, then 150 µL of reading buffer (PBS 1X, 1% de BSA) is added per well to read antigen-antibody reactions on Intelliflex (Luminex Corp., Austin, TX, USA). At least 100 events are read for each bead set, and the results are expressed as median fluorescence intensity (MFI) per 100 beads.

Assay performance determination

To determine the performance, we determined for each of the four proteins (NP, VP40, GPm and GPk), sensitivity, specificity, and accuracy with ROC curve analysis with XLSTAT (Addinsoft, Paris, France) implemented in Microsoft Excel software.

To define the assay consistence, we determined assay variabilities by calculating intra- and inter-assay variability. We determined the limits of quantification for both Glycoproteins (GPm and GPk) by using the two monoclonal antibodies as samples. We used the Belysa software (Merck-Millipore) to determine the upper and lower limits of quantification of the assay.

Results

Sensitivity, specificity and accuracy

We used the panel of 85 samples (43 positive and 42 negative) of known Ebola virus to determine the intrinsic performance of our test.

The results are summarized in the Table S.2.1 below and Figure S.2.1 and show excellent performances for all the four antigens, with accuracies (Area Under the Curve, AUC) comprised between 98.7% and 100%.

Table S2.1: Sensitivity, specificity, accuracy, and cut-off values of the four antigens used in the Luminex assay.

Antigens	Sensitivity (%)	Specificity (%)	Accuracy (%)	Cut-off (MFI)
**GPk**	100	100	100	169
**GPm**	97.7	92.9	98.7	1081
**NP**	100	100	100	590
**VP40**	100	100	100	869

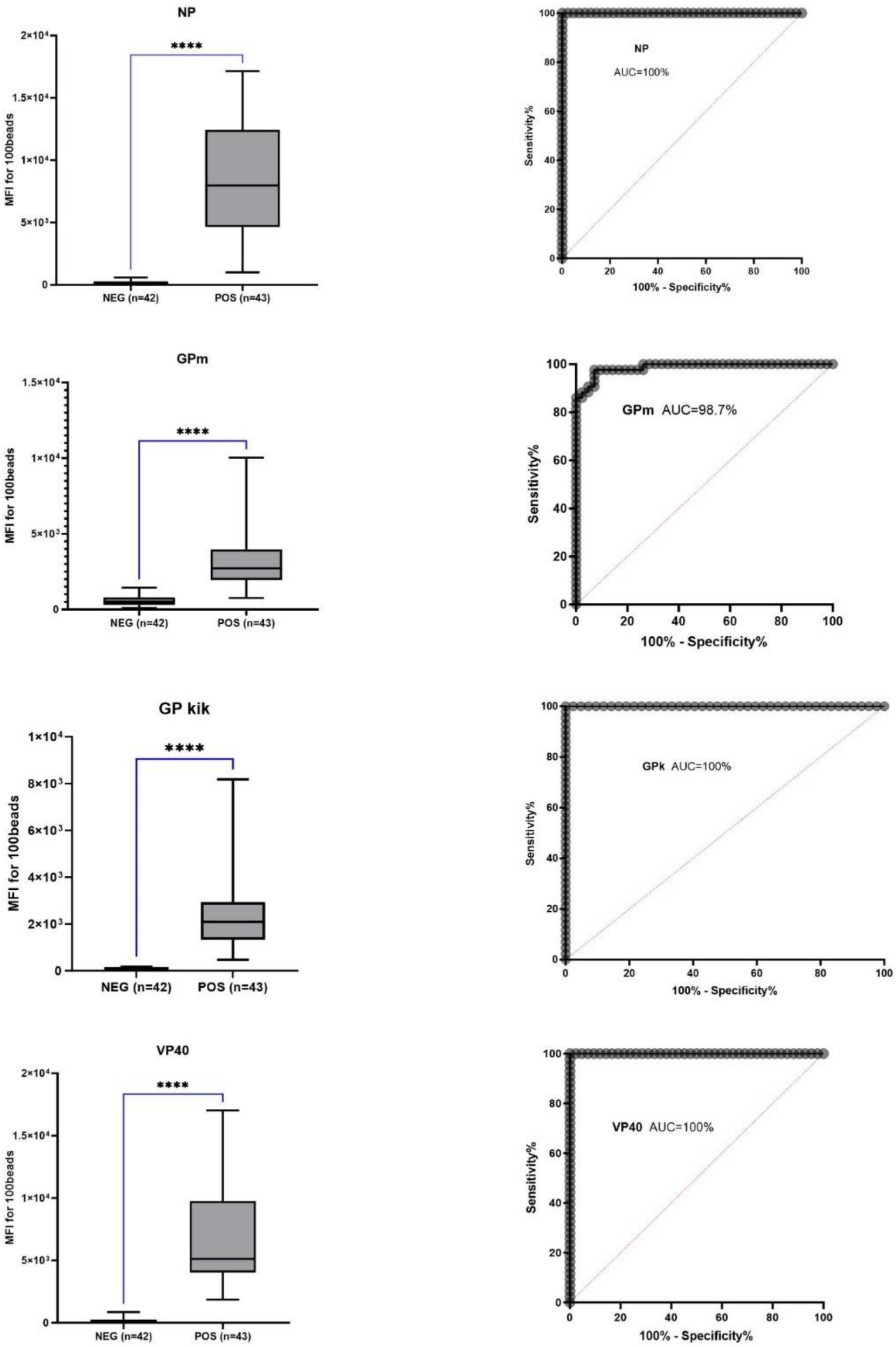

<u>Figure S2.1:</u> ROC curves of the four antigens used in the Luminex assay, together with boxplot comparison of negative and positive samples reactivities on the same four antigens. The statistical comparison was performed with the Mann-Whitney U-test. All p-values are highly significant ($<10^{-4}$). NP, Ebola virus nucleoprotein; GP, Ebola virus glycoprotein, VP40, Ebola virus 40kDa viral protein.

Intra-assay variability

To evaluate the robustness of our assay, we first assessed the intra-assay variability. To that end, we tested in the same run 12 replicates of each of the three references (i.e. reference Standard; HQC and LQC). From these 12 replicates, we calculated the coefficient of variation (CV) for each sample.

Results of this assessment are provided below, in percentage. It should be noted that for all the four antigens, the CV is below 20%.

Table S2.2: Intra-assay variability for the four antigens used, tested in 12 replicates within the same run.

	Intra-assay variability (%)			
	NP	GPmay	VP40	GPkikwit95
Ref Standard	11.38	10.26	12.69	13.51
HQC	12.86	11.62	14.40	12.02
LQC	18.76	9.90	13.25	11.72

Inter-assay variability
Next, we assessed the variability of the assay among plates ran on separate days by two different engineers. The same controls were used. For the reference standard, HQC (High Quality Control) and LQC (Low Quality Control), 19, 22, and 18 values were recorded, respectively. The results of the inter-assay variability are presented in Table S2.3 below and the showed that for all the four antigens the CVs are below 30%.

Table S2.3: inter-assay variability for the four antigens used, tested in 18-22 replicates in multiple runs.

	Inter-assay variability (%)			
	NP	GPmay	VP40	GPkikwit95
Ref standard	26.94	26.37	24.53	20.80
HQC	17.25	26.77	17.68	16.76
LQC	22.72	19.42	18.71	14.03

Determination of detection limits of antibodies against EBOV GP kikwit95 and Mayinga76.
To that end, we used two monoclonal antibodies raised against the two glycoproteins.
Figure S2.2 below illustrates such a titration curve for GPkikwit95 with the monoclonal antibody KZ52. The LLoQ is 112pg/ml, the MDD (Minimum Detectable Dose) is 89.26 pg/ml and the ULQ (Upper Limit of Quantification) is 10,000pg/ml.

GP zaire kikwit95new

Original Name	Current Name	ID	Units	Precision
GP zaire kikwit95new	GP zaire kikwit95new	45	pg/mL	2

Curve: Standard Curve Group 1 (4PL)

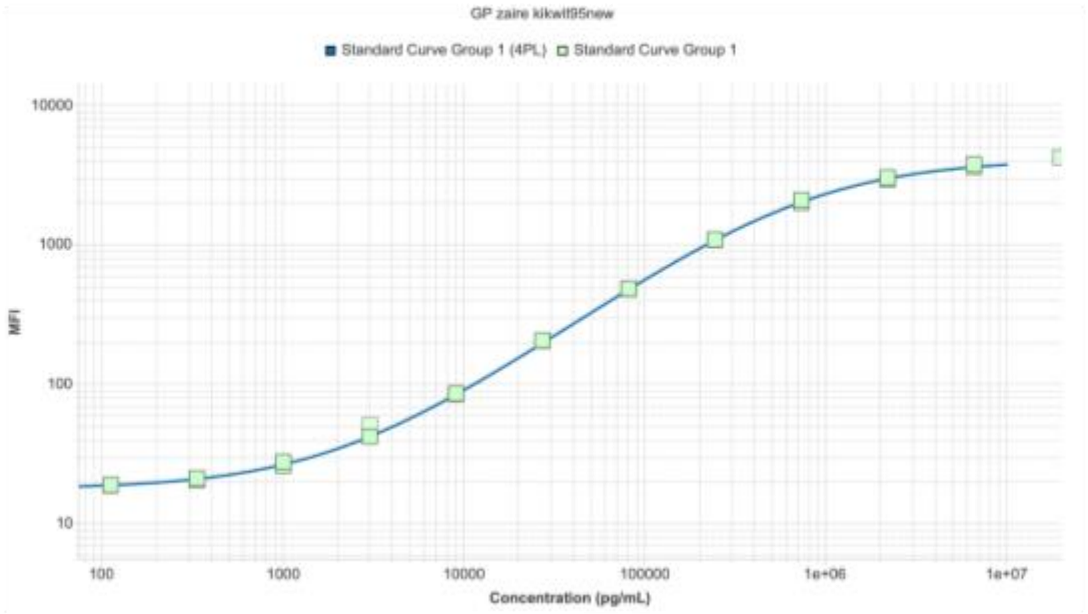

Fit	LLoQ	MDD	LoD	R Squared	Slope	Weighting	Equation
4PL	112.90	89.29	N/A	1.00	0.00	1/y^2	y = 17.63 + (4084.29 - 17.63)/(1 + (758122.39/x)^0.925921)

<u>Figure S2.2:</u> Titration curve of GPkikwit95 with the monoclonal antibody KZ52. Trend and limits for the MoAb LA63 against GPmayinga are similar.

3. ASSAY VALIDATION: QUANTERIX

**Test facility:**	Quanterix,<br>900 Middlesex Turnpike, Building 1, Billerica, MA 01821
**Sponsor:**	NIAID/Leidos
Sponsor’s Monitor:	Kevin Newell
**Dates:**	
Study initiation:	19 Aug 2022
Experimental start:	06 Sep 2022
Experimental completion:	29 Sep 2022
Final report:	26 Aug 2024
**Archive:**	Quanterix, 900 Middlesex Turnpike, Building 1, Billerica, MA 01821
**Personnel involved in the study:**	
Study Responsible:	Scott Van Arsdell, PhD<br>Quanterix, 900 Middlesex Turnpike, Building 1, Billerica, MA 01821<br><br>Susan Yan, PhD<br>Quanterix, 900 Middlesex Turnpike, Building 1, Billerica, MA 01821

Table of contents

Page

3. ASSAY VALIDATION: QUANTERIX **17**

Table of contents **18**

Table of figures **18**

Table of tables **19**

Abbreviations **20**

Brief summary **21**

1 Introduction **22**

2 Materials and methods **22**
- 2.1 Array 22
- 2.2 Materials 22
- 2.3 Methods 22
- 2.4 Preparation of controls 23

3 Archiving procedures **23**

4 Results **23**
- 4.1 Analytical Run Summary 23
- 4.2 Standard Curve Performance 23
- 4.3 Limit of Quantification with Sample Matrix 24
- 4.4 Intra-Assay Precision 24
- 4.5 Inter-Assay Precision 24
- 4.6 Dilutional Linearity/Parallelism 24
- 4.7 Spike and Recovery 25
- 4.8 Serum Sample Testing 25

5 Discussion **25**
- 5.1 Standard Curve Performance 25
- 5.2 Limit of Quantification with Sample Matrix 25
- 5.3 Intra-Assay Precision 26
- 5.4 Inter-Assay Precision 26
- 5.5 Dilutional Linearity/Parallelism 26
- 5.6 Spike and Recovery 26
- 5.7 Serum Sample Testing 26

6 Deviations **26**

7 Conclusion **26**

Figures and Tables **27**

Table of figures

Page

No table of figures entries found.

Table of tables

Page

Table 3.1 Assay Components 27

Table 3.2 Reagents for Manufacture of Quality Control Samples 27

Table 3.3 Target Concentrations for Validation Quality Control Samples 27

Table 3.4 Analytical Run Summary for EBOV GP Monomer Serology Assay Validation 28

Table 3.5 Typical Standard Curve for EBOV GP Monomer Serology Assay 28

Table 3.6 Analytical ULOQ, LLOQ and LOD for EBOV GP Monomer Serology Assay 29

Table 3.7 Standard Curve Intermediate Precision for EBOV GP Monomer Serology Assay 30

Table 3.8 Determination of Lower Limit of Quantification with Sample Matrix (EBOV GP Monomer)* 31

Table 3.9 Intra-assay Precision Data for EBOV GP Monomer Serology Assay (Plate 1) 33

Table 3.10 Intra-assay Precision Data for EBOV GP Monomer Serology Assay (Plate 2) 34

Table 3.11 Inter-assay Precision Data for EBOV GP Monomer Serology Assay 35

Table 3.12 Inter-assay Precision Data for EBOV GP Monomer Serology Assay 36

Table 3.13 Dilutional Linearity: Anti-EBOV GP Monomer Antibody Concentrations of Diluted Samples 37

Table 3.14 Dilutional Linearity of Diluted Samples 38

Table 3.15 Spike and Recovery: Anti-EBOV GP Monomer Antibody Concentrations of Spiked Samples* 39

Table 3.16 Spike and Recovery: % Recovery of Spiked Samples 40

Table 3.17 Spike and Recovery: Anti-EBOV GP Monomer Antibody Concentrations of Spiked Samples* 41

Table 3.18 Spike and Recovery: % Recovery of Spiked Samples 42

Table 3.19 Concentration of anti-EBOV GP Monomer Antibodies in IRF Samples* 43

Table 3.20 Concentration of anti-EBOV GP Monomer Antibodies in IRF Samples* 44

Abbreviations

**Abbreviation**	**Definition**
% CV	Percent coefficient of variation
ALQ	Above Limit of Quantification
BAR	Biotinylated Antibody Reagent
BLD	Below Limit of Detection
BLQ	Below Limit of Quantification
CCD	Couple-Charged Device
EBOV	Ebola Virus
ELISA	Enzyme Linked Immunosorbent Assay
HQC	High Quality Control
HRP	Horseradish Peroxidases
IV	Intensity Value
LLOQ	Lower Limit of Quantification
LOD	Limit of Detection
LQC	Low Quality Control
mg	milligram
mL	milliliter
MRD	Minimum required dilution
MQC	Medium Quality Control
µL	Microliter
pg	picogram
QC	Quality Control
% RE	Percent relative error
SA-HRP	Streptavidin HRP
SD	Standard Deviation
SOP	Standard Operating Procedure
ULOQ	Upper Limit of Quantification
VHQC	Very High Quality Control
VLQC	Very Low Quality Control

Brief summary

The SP-X platform is a multiplex sandwich enzyme-linked immunosorbent assay (ELISA) in a planar, plate-based array format, for the quantitative measurement of secreted proteins in serum, plasma, tissue culture supernatants and other matrices. The SP-X platform was used in this project to develop and validate a serology assay for the detection of IgG antibodies to EBOV GP monomer protein in human serum. Each well of the microplate is pre-spotted with GP monomer protein, which binds to anti-GP monomer antibodies in the standards, controls and samples added to the plate. After unbound antibodies and serum components are washed away, a biotinylated anti-IgG detection antibody is added that binds to IgG antibodies bound to the GP monomer target protein. After washing away excess detection antibody, streptavidin-horseradish peroxidase (SA-HRP) is added. The HRP enzyme conjugate subsequently reacts with the chemiluminescent substrate to produce a luminescent signal that is detected using the SP-X CCD imaging and analysis system. The amount of signal produced is proportional to the amount of anti-GP monomer antibody in the original standard or sample. Customized SP-X software uses a five-parameter curve fit to back calculate unknowns using results extrapolated from the corresponding standard curve.

The objective of this study was to validate a planar serology immunoassay for the detection of IgG antibodies to EBOV GP monomer protein in human serum samples. Validation studies included an assessment of standard curve performance, determination of the limit of quantification (LLOQ) with human serum samples, intra-assay and inter-assay precision with spiked normal human serum samples, dilutional linearity with human serum samples, spike and recovery with human serum samples, and a reference range study with serum samples from patients who have been vaccinated with the EBOV vaccine. The results of the study indicate that the GP monomer serology immunoassay is valid and suitable for clinical sample analysis.

1 Introduction

The SP-X platform is a multiplex sandwich enzyme-linked immunosorbent assay (ELISA) in a planar, plate-based array format, for the quantitative measurement of secreted proteins in serum, plasma, tissue culture supernatants and other matrices. The SP-X platform was used in this project to develop and validate a serology assay for the detection of IgG antibodies to EBOV GP monomer protein in human serum. Each well of the microplate is pre-spotted with GP monomer protein, which binds to anti-GP monomer antibodies in the standards, controls and samples added to the plate. After unbound antibodies and serum components are washed away, a biotinylated anti-IgG detection antibody is added that binds to IgG antibodies bound to the GP monomer target protein. After washing away excess detection antibody, streptavidin-horseradish peroxidase (SA-HRP) is added. The HRP enzyme conjugate subsequently reacts with the chemiluminescent substrate to produce a luminescent signal that is detected using the SP-X CCD imaging and analysis system. The amount of signal produced is proportional to the amount of anti-GP monomer antibody in the original standard or sample. Customized SP-X software uses a five-parameter curve fit to back calculate unknowns using results extrapolated from the corresponding standard curve.

The objective of this study was to validate a planar serology immunoassay for the detection of IgG antibodies to EBOV GP monomer protein in human serum samples. Validation studies included an assessment of standard curve performance, determination of the limit of quantification (LLOQ) with human serum samples, intra-assay and inter-assay precision with spiked normal human serum samples, dilutional linearity with human serum samples, spike and recovery with human serum samples, and a reference range study with serum samples from patients who have been vaccinated with the EBOV vaccine. The results of the study indicate that the GP monomer serology immunoassay is valid and suitable for clinical sample analysis.

2 Materials and methods

2.1 Array

A planar serology immunoassay array was constructed for detection of the following target antibody:

EBOV GP monomer IgG

2.2 Materials

Each assay consisted of a 96-well plate custom arrayed with GP monomer protein, a reference standard serum, sample diluent containing 0.1% sodium azide, biotinylated antibody reagent, SA-HRP reagent, stable peroxide solution, luminol enhancer solution, and wash buffer. The lot numbers and expiration dates for the critical assay components are presented in Table 3.1. Plates were read using the SP-X CCD camera imaging and analysis system. Images were analysed using SP-X software and data analysis was completed and summarized using Microsoft® Excel.

2.3 Methods

Prior to running assays, all reagents were brought to room temperature. Plates were washed using the Quanterix plate washer SP-X program. Diluted samples, standards and controls were incubated for two hours on the arrayed plates. All incubations were performed at ambient temperature with shaking at 450 rpm. Plates were washed using the Quanterix plate washer SP-X program before adding the biotinylated detection antibody to each well. After incubating with detection antibody for 30 minutes, plates were washed and incubated for 30 minutes with streptavidin-horseradish

peroxidase. Plates were again washed before adding the chemiluminescent substrate. The plates were then imaged using the SP-X imaging system and data was analysed using the SP-X software. Concentrations of all unknown samples were back calculated using results extrapolated from the corresponding standard curve. A five-parameter curve fit was used for all standard curves.

2.4 Preparation of controls

Control samples corresponding to very high, high, medium, low, and very low concentration levels (VHQC, HQC, MQC, LQC, VLQC) were created for the GP monomer serology assay. A serum sample with high levels of anti-EBOV GP monomer antibodies (lot number PREP0001, received from NIAID) was spiked into two normal human serum samples (BRH233209 and HMN403957) at five concentration levels (VHQC, HQC, MQC, LQC, VLQC) for each sample to create ten individual control samples for the validation. Two intermediate controls for each concentration level (pre-VHQC-1, pre-VHQC-2, pre-HQC-1, pre-HQC-2, pre-MQC-1, pre-MQC-2, pre-LQC-1, pre-LQC2, pre-VLQC-1 and pre-VLQC-2) were manufactured, aliquoted into individual vials and stored at -80°C. The intermediate controls were diluted to the final target concentrations for VHQC-1, VHQC-2, HQC-1, HQC-2, MQC-1, MQC-2, LQC-1, LQC2, VLQC-1 and VLQC-2 on the day of the assay. The lot numbers and expiration dates of the reagents used to manufacture quality control samples are presented in Table 3.2. The final target concentrations for the quality control samples are presented in Table 3.

3 Archiving procedures

Data and the report will be stored at Quanterix, 900 Middlesex Turnpike, Building 1, Billerica, MA 01821. Electronic data are backed up daily. All documents and data related to the assay validations and sample testing service will be stored for five years by Quanterix.

4 Results

4.1 Analytical Run Summary

Validation of the EBOV GP monomer planar serology immunoassay was performed over 12 analytical runs presented in Table 3.4. Twelve analytical runs were accepted.

4.2 Standard Curve Performance

The standard curve assay range for the EBOV GP monomer serology assay was determined during development and was selected to optimize sensitivity and linearity. Standard curves were run in assay Diluent 22. A minimum of six standard curves were evaluated over the course of the validation. Seven non-zero calibrators in triplicate within the anticipated range were included for a concentration-response relationship fit using the five-parameter logistic function. The standard curve was generated by serially diluting standard calibrator 1 with four-fold intervals.
Standard curve performance is acceptable if the CV of the mean signal (integrated density value) for standards 1-5 is < 15%, the CV of the mean signal (integrated density value) for standards 6 and 7 is <20%, the percent recovery for the calculated back fit concentration for standards 1-6 is 80 – 120% and the percent recovery for the calculated back fit concentration for standard 7 is 75 – 125%.
The following equations were used to calculate % CV and % RE.
% CV = standard deviation ÷ mean signal of duplicate wells x 100
% Recovery = measured concentration ÷ nominal concentration x 100
% RE = (measured concentration – nominal concentration) ÷ nominal concentration x 100
The upper and lower limits of quantification (ULOQ and LLOQ) for each analyte was established based on the accuracy and repeatability of the high and low reference standards. The limit of

detection (LOD) was calculated by adding 2.55 standard deviations of signal to the mean signal of the replicates of the zero standards and determining the resulting concentration from the standard curve. The Lower Limit of Quantification (LLOQ) of the assay was defined as the lowest calibration standard with a back-calculated concentration CV of less than 20% and back-calculated concentration relative error of less than 25%. The Upper Limit of Quantification (ULOQ) of the assay was defined as the highest calibration standard with a back-calculated concentration of less than 20% and back-calculated concentration relative error of less than 20%.
A typical standard curve for the EBOV GP monomer serology assay is presented in Table 3.5. The analytical ULOQ, LLOQ and LOD for the EBOV GP monomer serology assay is presented in Table 3.6. The LOD shown in Table 3.6 is the average LOD value from 10 calibration curves. The backfit concentrations of each calibrator for each EBOV GP monomer assay run of the validation (n = 12 runs) is presented in Table 3.7. The mean backfit value, standard deviation, % CV, % nominal and % RE are also presented.

4.3 Limit of Quantification with Sample Matrix

The LLOQ of the EBOV GP monomer serology assay was also determined with sample matrix. Four serum samples from NIAID with levels of anti-EBOV GP monomer antibodies that are above the LLOQ determined with the calibration standards were used for this analysis. Each sample was diluted 1:4,000, 1:8,000, 1:16,000, 1:32,000, 1:64,000, 1:128,000, 1:256,000 and 1:512,000 in sample diluent and then run on the assay. The LLOQ for sample matrix was defined as the lowest analyte concentration that can quantified in serum with acceptable precision (+/- 25%). The concentration data for each sample replicate for each sample dilution is presented in Table 3.8. The LLOQ values determined with sample matrix for each sample are also shown in Table 3.8. The average LLOQ value determined with sample matrix was 0.002 EU/ml.

4.4 Intra-Assay Precision

Control samples VHQC-1, VHQC-2, HQC-1, HQC-2, MQC-1, MQC-2, LQC-1, LQC2, VLQC-1 and VLQC-2 were created as described in Section 2.4. Intra-assay precision was evaluated by running 16 replicates each of VHQC-1, HQC-1, MQC-1, LQC-1 and VLQC-1 on one plate and 16 replicates each of VHQC-2, HQC-2, MQC-2, LQC-2 and VLQC-2 on a second plate. The % CV of back-calculated protein concentration was calculated for each control sample. The intra-assay precision was considered acceptable if % CV was ≤25%. Intra-assay precision data is presented in Table 3.9 and Table 3.10.

4.5 Inter-Assay Precision

Inter-assay precision was evaluated by running duplicates of VHQC-1, VHQC-2, HQC-1, HQC-2, MQC-1, MQC-2, LQC-1, LQC2, VLQC-1 and VLQC-2 on at least four assay plates on four separate days, using at least two operators and calculating % CV of back-calculated protein concentration for each control sample. The inter-assay precision was considered acceptable if % CV was ≤25%. Inter-assay precision data is presented in Table 3.11 and Table 3.12.

4.6 Dilutional Linearity/Parallelism

Dilution and recovery experiments were performed to assess the dilutional linearity of the EBOV GP monomer serology assay. Five serum samples from NIAID with high levels of anti-EBOV GP monomer antibodies were used for this analysis. Each sample was diluted 1:2,000, 1:4,000, 1:8,000, 1:16,000 and 1:32,000 in sample diluent and then run on the assay. The measured concentration of each sample dilution was multiplied by the dilution factor and compared to the

concentration of the 1:4,000 dilution. Recoveries of each of the dilutions was calculated as a percent recovery of protein relative to the 1:4,000 dilution. The 1:8,000 sample dilution was also used as a reference for calculating % recovery. Dilutional linearity is considered acceptable if recovery is ±30% of the reference concentration value. The antibody concentrations of the diluted samples are shown in Table 3.13 and the percent recoveries for each sample are shown in Table 3.14.

4.7 Spike and Recovery

Spike and recovery experiments were performed to assess serum matrix effects and the accuracy of the method in serum samples. Serum samples received from NIAID were spiked with a serum sample containing high levels of anti-EBOV GP monomer antibodies (lot number PREP0001, received from NIAID). Ten individual serum samples were diluted at 1:2000 with sample diluent and spiked at very high, medium, low and very low concentrations of anti-EBOV GP monomer antibodies spanning the calibration range of the assay. The very high spike was targeted to fall between standards 1 and 2, the high spike between standards 2 and 3, the medium spike between standards 3 and 4, the low spike between standards 4 and 5 and the very low spike between standards 5 and 6. Each serum sample was also assayed without a spike to measure endogenous levels for use in recovery calculations. The final sample dilution after the spike solution was added was 1:4,000. The measured value from the un-spiked matrix-matched sample is subtracted from the measured values of each of the spiked samples. The net value was compared to the measured value of the five concentrations of protein spiked into the control diluent. The sample matrix effect is represented as a percent recovery of expected protein concentration. Percent recovery was calculated using the following equation:
% Recovery = (spiked sample concentration – endogenous) ÷ control spike concentration x 100
Spike and recovery is considered acceptable if the recovery of each analyte in ≥ 5/7 samples is within ±30% of the control spike concentration. The measured concentrations of anti-EBOV antibodies in spiked samples are presented in Table 3.15 and Table 3.17. The percent recovery for each of the spiked samples is presented in Table 3.16 and Table 3.18.

4.8 Serum Sample Testing

Sixty serum samples from vaccinated individuals received from the IRF were analysed with the EBOV GP monomer serology assay. Samples were diluted 1:4,000 with sample diluent and then run on the assay. The measured concentration of anti-EBOV GP monomer antibodies for each sample is presented in Table 3.19 and Table 3.20.

5 Discussion

5.1 Standard Curve Performance

All standard curves for the validation assay runs met the specifications for precision and accuracy described in Section 4.2. The backfit concentrations of each calibrator for each assay run (n = 12) are presented in Table 3.7. The mean backfit value, standard deviation, % CV, % nominal, and % RE are acceptable for all calibration curves run for the validation study.

5.2 Limit of Quantification with Sample Matrix

The LLOQ for sample matrix was defined as the lowest analyte concentration that can be quantified in serum with acceptable precision (+/- 25%). The LLOQ values determined with sample matrix for

the EBOV GP monomer serology assay are shown in Table 3.8. The average LLOQ value determined with sample matrix was 0.002 EU/ml.

5.3 Intra-Assay Precision

Intra-assay precision data is presented in Table 3.9 and Table 3.10. The intra-assay precision is considered acceptable as the % CV for all quality control samples for all assays was ≤25%.

5.4 Inter-Assay Precision

Inter-assay precision data is presented in Table 3.11 and Table 3.12. The inter-assay precision is considered acceptable as the % CV for all quality control samples for all assays was ≤25%.

5.5 Dilutional Linearity/Parallelism

Dilution and recovery experiments were performed to assess the dilutional linearity of the EBOV GP monomer serology assay. The antibody concentrations of the diluted samples are shown in Table 3.13 and the percent recoveries for each of the five samples are shown in Table 3.14. Dilutional linearity is considered acceptable if recovery of at least 2/3 (67%) of the dilution comparisons for 80% of the individual serum samples is within 70-130%. Dilutional linearity was acceptable for the five serum samples analysed whether the 1:4,000 or 1:8,000 dilution was used as the reference concentration (Table 3.14).

5.6 Spike and Recovery

The results for spike and recovery analysis are shown in Table 3.15-Table 3.18. The measured concentrations of anti-EBOV antibodies in spiked samples are presented in Table 3.15 and Table 3.17. The percent recovery for each of the spiked samples is presented in Table 3.16 and Table 3.18. Spike recovery is considered acceptable if recovery of 4/5 (80%) of the spike levels for 80% of the individual samples is within 70-130% of the control diluent spike. Spike recovery was acceptable for all ten serum samples at all five spike levels.

5.7 Serum Sample Testing

Sixty serum samples from vaccinated individuals received from the IRF were analysed with the EBOV GP monomer serology assay. The measured concentrations of anti-EBOV GP monomer antibodies for each sample are presented in Table 3.19 and Table 3.20. The measured concentration of anti-EBOV GP monomer antibodies was within the quantitative range of the assay for 87% of the samples. Eight samples were below the functional LLOQ (19.6 EU/ml). The % CV for sample replicates was <25% for all samples tested.

6 Deviations

There were no deviations for this study.

7 Conclusion

The validation was approved, and the assays can be used for clinical sample analysis.

Figures and Tables

Table 3.1: Assay Components

Component	Lot Number	Expiration Date
GP Monomer Plate	063022P	30-Jun-2027
Biotinylated Detection Antibody Reagent	080122SY	1-Aug-2025
Reference Standard Serum	BMIZAIRE 108	30-Sep-2023
Diluent 22	319097	31-Oct-2022
Diluent 22	319115	7-Nov-2022
Streptavidin HRP	319747	5-May-2025

Table 3.2: Reagents for the Manufacture of Quality Control Samples

Reagent	Lot Number	Expiration Date
High Titer Serum	PREP0001	2-Aug-2024
Normal Human Serum	BRH233209	1-Sep-2023
Normal Human Serum	HMN403957	1-Sep-2023

Table 3.3: Target Concentrations for Validation Quality Control Samples

Sample	Target Concentration (EU/ml)
VHQC	10
HQC	2.5
MQC	0.625
LQC	0.156
VLQC	0.039

Table 3.4: Analytical Run Summary for the EBOV GP Monomer Serology Assay Validation

Assay Run	Date	Plate Identity	Status	Tables
1	6-Sep-2022	serum dil linearity ebovgpmono 063022p-012 as0073 220906 151036.sdf	Accepted	7,11,12,13,14
2	7-Sep-2022	plate1 loq ebovgpmono 063022p-013 as0073 220907 150656.sdf	Accepted	7,8,11,12
3	7-Sep-2022	plate2 loq ebovgpmono 063022p-014 as0073 220907 151300.sdf	Accepted	7,8,11,12
4	8-Sep-2022	spk-rvy1 ebovgpmono 063022p-015 as0073 220908 144235.sdf	Accepted	7,11,12,15,16
5	9-Sep-2022	spk-rvy2 ebovgpmono 063022p-016 as0073 220909 151958.sdf	Accepted	7,11,12,15,16,17,18
6	9-Sep-2022	spk-rvy3 ebovgpmono 063022p-017 as0073 220909 152629.sdf	Accepted	7,11,12,17,18
7	12-Sep-2022	plate1 intra-precision ebovgpmono 063022p-018 as0073 220912 145455.sdf	Accepted	7,9,11,12
8	12-Sep-2022	plate2 intra-precision ebovgpmono 063022p-019 as0073 220912 150142.sdf	Accepted	7,10
9	13-Sep-2022	spk-rvy4 ebovgpmono 063022p-020 as0073 220913 160135.sdf	Accepted	7,11,12,17,18
10	27-Sep-2022	ebovgp az 1 1 ebovgpmono 063022p-023 as0073 220927 151153.sdf	Accepted	5,7,11,12
11	29-Sep-2022	plate3 irf13-45 serum samples ebovgpmono 063022p-025 as0073 220929 173534.sdf	Accepted	7,19,20
12	29-Sep-2022	plate4 irf61-95 serum samples ebovgpmono 063022p-026 as0073 220929 174221.sdf	Accepted	7,19,20

Table 3.5: Typical Standard Curve for the EBOV GP Monomer Serology Assay

Calibrator	GP Monomer (EU/ml)	Average Signal (IV)	SD	% CV	% RE
1	20.0	4638347	153965	3.3	4.4
2	5.00	1985204	167534	8.4	1.1
3	1.25	580815	9260	1.6	7.3
4	0.313	164173	5689	3.5	5.1
5	0.078	39289	1175	3.0	8
6	0.0195	8561	976	11.4	1.5
7	0.0049	1733	204	11.8	20.7
8	0.0000	331	34	10.2	NA

Table 3.6: Analytical ULOQ, LLOQ, and LOD for the EBOV GP Monomer Serology Assay

GP Monomer	EU/ml
ULOQ	20.0
LLOQ	0.0049
LOD	0.0009

Table 3.7: Standard Curve Intermediate Precision for the EBOV GP Monomer Serology Assay

Plate	Assay Run	Assay Date	Std1 20.0 EU/ml	Std2 5.00 EU/ml	Std3 1.25 EU/ml	Std4 0.313 EU/ml	Std5 0.078 EU/ml	Std6 0.020 EU/ml	Std7 0.005 EU/ml
serum dil linearity ebovgpmono 063022p-012 as0073 220906 151036.sdf	1	6-Sep-2022	20.5	4.64	1.29	0.327	0.085	0.018	0.004
plate1 loq ebovgpmono 063022p-013 as0073 220907 150656.sdf	2	7-Sep-2022	20.0	4.59	1.25	0.338	0.082	0.019	0.004
plate2 loq ebovgpmono 063022p-014 as0073 220907 151300.sdf	3	7-Sep-2022	20.4	4.60	1.27	0.319	0.084	0.020	0.004
spk-rvy1 ebovgpmono 063022p-015 as0073 220908 144235.sdf	4	8-Sep-2022	19.9	4.83	1.26	0.317	0.081	0.016	*
spk-rvy2 ebovgpmono 063022p-016 as0073 220909 151958.sdf	5	9-Sep-2022	20.4	4.79	1.27	0.340	0.083	0.018	0.004
spk-rvy3 ebovgpmono 063022p-017 as0073 220909 152629.sdf	6	9-Sep-2022	20.3	5.02	1.29	0.314	0.081	0.019	0.004
plate1 intra-precision ebovgpmono 063022p-018 as0073 220912 145455.sdf	7	12-Sep-2022	20.1	4.76	1.29	0.318	0.081	0.019	0.004
plate2 intra-precision ebovgpmono 063022p-019 as0073 220912 150142.sdf	8	12-Sep-2022	21.3	4.77	1.25	0.316	0.083	0.020	0.004
spk-rvy4 ebovgpmono 063022p-020 as0073 220913 160135.sdf	9	13-Sep-2022	21.2	4.76	1.20	0.303	0.082	0.020	0.004
ebovgp az 1 1 ebovgpmono 063022p-023 as0073 220927 151153.sdf	10	27-Sep-2022	20.9	4.94	1.16	0.329	0.084	0.020	0.004
plate3 irf13-45 serum samples ebovgpmono 063022p-025 as0073 220929 173534.sdf	11	29-Sep-2022	21.6	4.76	1.19	0.321	0.083	0.023	0.004
plate4 irf61-95 serum samples ebovgpmono 063022p-026 as0073 220929 174221.sdf	12	29-Sep-2022	19.4	4.99	1.16	0.342	0.084	0.021	0.004
Mean			20.5	4.79	1.24	0.324	0.083	0.019	0.004
SD			0.6	0.14	0.05	0.012	0.001	0.002	0.0003
% CV			3.1	2.95	4.09	3.6	1.8	8.4	7.0
% Nominal			102.5	95.8	99.1	103.6	105.9	99.8	80.3
% RE			2.5	-4.2	-0.9	3.6	5.9	-0.2	-19.7
N			12	12	12	12	12	12	11

*Cell without data indicates calibration point removed which did not meet acceptance criteria.

Table 3.8: Determination of Lower Limit of Quantification with Sample Matrix (EBOV GP Monomer)*

Sample	Dilution Factor	Replicate 1 (EU/ml)	Replicate 2 (EU/ml)	Replicate 3 (EU/ml)	Average (EU/ml)	SD	%CV	LLOQ
PREVAIL 9	4,000	0.108	0.108	0.101	0.105	0.004	3.8	0.001
	8,000	0.049	0.049	0.048	0.048	0.0005	1.0	
	16,000	0.027	0.024	0.022	0.024	0.002	9.3	
	32,000	0.010	0.011	0.010	0.010	0.000	2.9	
	64,000	0.005	**0.004**	0.006	**0.005**	0.001	19.3	
	128,000	**0.002**	**0.002**	**0.003**	**0.003**	0.0001	5.5	
	256,000	**0.002**	**0.002**	**0.002**	**0.002**	0.0002	10.7	
	512,000	**0.001**	**0.001**	**0.002**	**0.001**	0.0001	10.6	
PREVAIL 27	4,000	0.253	0.362	0.324	0.313	0.055	17.6	0.002
	8,000	0.143	0.135	0.143	0.140	0.005	3.5	
	16,000	0.056	0.067	0.072	0.065	0.008	12.9	
	32,000	0.028	0.032	0.033	0.031	0.002	7.5	
	64,000	0.013	0.013	0.014	0.013	0.001	5.4	
	128,000	0.008	0.007	0.007	0.007	0.001	10.1	
	256,000	**0.004**	**0.004**	**0.003**	**0.004**	0.0005	13.7	
	512,000	**0.002**	**0.002**	**0.002**	**0.002**	0.0000	1.1	
PREVAIL 79	4,000	0.267	0.271	0.278	0.272	0.006	2.1	0.002
	8,000	0.131	0.130	0.131	0.131	0.001	0.5	
	16,000	0.067	0.065	0.065	0.066	0.001	1.9	
	32,000	0.031	0.032	0.030	0.031	0.001	2.9	
	64,000	0.015	0.014	0.014	0.014	0.001	4.2	
	128,000	0.007	0.007	0.007	0.007	0.0004	6.0	
	256,000	**0.003**	**0.003**	**0.003**	**0.003**	0.0003	9.8	
	512,000	**0.002**	**0.002**	**0.002**	**0.002**	0.0001	5.6	
PREVAIL 91	4,000	0.290	0.301	0.297	0.296	0.005	1.8	0.002
	8,000	0.146	0.144	0.142	0.144	0.002	1.4	
	16,000	0.072	0.069	0.072	0.071	0.002	2.2	

	32,000	0.035	0.034	0.034	0.035	0.0004	1.0	
	64,000	0.015	0.015	0.016	0.015	0.0003	1.7	
	128,000	0.008	0.007	0.007	0.007	0.0003	3.9	
	256,000	**0.004**	**0.003**	**0.004**	**0.004**	0.0004	9.9	
	512,000	**0.002**	**0.002**	**0.002**	**0.002**	0.0001	4.5	

*Bold values are below lowest calibration standard

Table 3.9: Intra-assay Precision Data for the EBOV GP Monomer Serology Assay (Plate 1)

Replicate	VHQC-1 (EU/ml)	HQC-1 (EU/ml)	MQC-1 (EU/ml)	LQC-1 (EU/ml)	VLQC-1 (EU/ml)
1	9.03	2.09	0.517	0.163	0.069
2	8.25	1.91	0.514	0.156	0.071
3	8.48	1.92	0.492	0.149	0.074
4	8.60	1.98	0.491	0.154	0.071
5	8.33	2.00	0.476	0.150	0.073
6	8.93	1.83	0.554	0.157	0.074
7	8.45	2.10	0.560	0.155	0.069
8	9.24	2.12	0.562	0.170	0.080
9	8.57	2.17	0.564	0.155	0.076
10	7.79	1.95	0.507	0.149	0.089
11	7.89	1.95	0.484	0.142	0.073
12	7.49	1.87	0.507	0.152	0.072
13	8.49	1.91	0.482	0.160	0.084
14	7.83	2.00	0.489	0.156	0.081
15	9.07	2.00	0.494	0.144	0.080
16	8.95	2.21	0.562	0.145	0.079
Average	8.46	2.00	0.516	0.154	0.076
SD	0.514	0.109	0.033	0.007	0.006
%CV	6.1	5.5	6.4	4.7	7.4

Table 3.10: Intra-assay Precision Data for the EBOV GP Monomer Serology Assay (Plate 2)

Replicate	VHQC-2 (EU/ml)	HQC-2 (EU/ml)	MQC-2 (EU/ml)	LQC-2 (EU/ml)	VLQC-2 (EU/ml)
1	8.40	1.93	0.456	0.143	0.041
2	7.34	1.65	0.465	0.126	0.041
3	7.71	1.69	0.456	0.133	0.045
4	7.11	1.73	0.448	0.140	0.044
5	7.27	1.70	0.437	0.131	0.042
6	7.83	1.58	0.516	0.134	0.042
7	7.64	1.78	0.518	0.136	0.039
8	8.19	1.98	0.559	0.146	0.048
9	7.68	1.88	0.524	0.130	0.042
10	6.66	1.70	0.490	0.123	0.050
11	6.77	1.59	0.454	0.132	0.044
12	6.47	1.59	0.461	0.138	0.044
13	6.59	1.60	0.427	0.136	0.044
14	6.67	1.73	0.462	0.132	0.047
15	8.53	1.81	0.479	0.124	0.043
16	8.13	2.01	0.522	0.122	0.048
Average	7.44	1.75	0.480	0.133	0.044
SD	0.680	0.142	0.038	0.007	0.003
%CV	9.1	8.1	7.9	5.1	6.7

Table 3.11: Inter-assay Precision Data for the EBOV GP Monomer Serology Assay

Operator	Day	Plate	VHQC-1 (EU/ml)	HQC-1 (EU/ml)	MQC-1 (EU/ml)	LQC-1 (EU/ml)	VLQC-1 (EU/ml)
1	1	1	8.32	2.10	0.538	0.155	0.085
1	2	1	10.5	2.33	0.575	0.190	0.116
1	2	2	8.14	2.08	0.536	0.178	0.101
1	3	1	8.59	2.51	0.611	0.170	0.077
1	4	1	9.16	2.00	0.551	0.184	0.103
1	4	2	8.42	2.37	0.573	0.174	0.096
1	5	1	8.46	2.00	0.516	0.154	0.076
1	6	1	9.32	2.27	0.541	0.185	0.102
2	7	1	8.99	1.88	0.503	0.197	0.128
Average			8.88	2.17	0.549	0.176	0.098
SD			0.734	0.208	0.033	0.015	0.017
%CV			8.3	9.6	6.0	8.4	17.7

Table 3.12: Inter-assay Precision Data for the EBOV GP Monomer Serology Assay

Operator	Day	Plate	VHQC-2 (EU/ml)	HQC-2 (EU/ml)	MQC-2 (EU/ml)	LQC-2 (EU/ml)	VLQC-2 (EU/ml)
1	1	1	8.32	2.17	0.558	0.148	0.049
1	2	1	9.24	2.04	0.574	0.177	0.062
1	2	2	8.21	1.99	0.497	0.152	0.055
1	3	1	7.95	2.22	0.540	0.138	0.039
1	4	1	8.17	2.15	0.565	0.165	0.057
1	4	2	7.57	2.23	0.556	0.154	0.055
1	5	1	7.44	1.75	0.480	0.133	0.044
1	6	1	8.67	2.08	0.509	0.162	0.070
2	7	1	7.26	1.98	0.574	0.182	0.086
Average			8.09	2.07	0.539	0.157	0.058
SD			0.626	0.151	0.035	0.016	0.014
%CV			7.7	7.3	6.6	10.5	24.7

Table 3.13: Dilutional Linearity: Anti-EBOV GP Monomer Antibody Concentrations of Diluted Samples

Sample	Dilution Factor	GP Monomer (EU/ml)
PREVAIL 9	2,000	389
	4,000	347
	8,000	363
	16,000	346
	32,000	311
PREVAIL 10	2,000	140
	4,000	171
	8,000	159
	16,000	128
	32,000	105
PREVAIL 14	2,000	1,774
	4,000	1,748
	8,000	1,744
	16,000	2,014
	32,000	2,090
PREVAIL 17	2,000	15,820
	4,000	18,260
	8,000	13,450
	16,000	14,280
	32,000	13,580
PREVAIL 85	2,000	27,010
	4,000	27,570
	8,000	30,690
	16,000	37,500
	32,000	34,220

Table 3.14: Dilutional Linearity of Diluted Samples

Sample	Dilution Factor	% Recovery 1:4,000	% Recovery 1:8,000
PREVAIL 9	2,000	112	107
	4,000	100	96
	8,000	105	100
	16,000	100	95
	32,000	90	86
PREVAIL 10	2,000	82	88
	4,000	100	107
	8,000	93	100
	16,000	75	80
	32,000	62	66
PREVAIL 14	2,000	101	102
	4,000	100	100
	8,000	100	100
	16,000	115	115
	32,000	120	120
PREVAIL 17	2,000	87	118
	4,000	100	136
	8,000	74	100
	16,000	78	106
	32,000	74	101
PREVAIL 85	2,000	98	88
	4,000	100	90
	8,000	111	100
	16,000	136	122
	32,000	124	112

Table 3.15: Spike and Recovery: Anti-EBOV GP Monomer Antibody Concentrations of Spiked Samples*

Sample	Spike	GP Monomer (EU/ml)
Prevail 2	Very High	11.5
	High	2.64
	Medium	0.734
	Low	0.185
	Very Low	0.043
	None	**0.001**
Prevail 3	Very High	10.3
	High	2.74
	Medium	0.722
	Low	0.174
	Very Low	0.039
	None	**0.0002**
Prevail 6	Very High	11.8
	High	2.73
	Medium	0.742
	Low	0.190
	Very Low	0.040
	None	**0.0002**
Prevail 7	Very High	11.0
	High	2.49
	Medium	0.633
	Low	0.163
	Very Low	0.037
	None	**0.0004**
Prevail 8	Very High	9.11
	High	2.34
	Medium	0.548
	Low	0.153
	Very Low	0.039
	None	**0.0002**

*Bold values <LLOQ

Table 3.16: Spike and Recovery: Percent Recovery of Spiked Samples

Sample	Spike	% Recovery
Prevail 2	Very High	100
	High	87
	Medium	91
	Low	86
	Very Low	100
Prevail 3	Very High	90
	High	90
	Medium	89
	Low	81
	Very Low	91
Prevail 6	Very High	103
	High	90
	Medium	92
	Low	89
	Very Low	94
Prevail 7	Very High	105
	High	88
	Medium	96
	Low	87
	Very Low	93
Prevail 8	Very High	87
	High	83
	Medium	84
	Low	82
	Very Low	98

Table 3.17: Spike and Recovery: Anti-EBOV GP Monomer Antibody Concentrations of Spiked Samples*

Sample	Spike	GP Monomer (EU/ml)
Prevail 11	Very High	10.0
	High	2.38
	Medium	0.603
	Low	0.161
	Very Low	0.038
	None	**0.001**
Prevail 12	Very High	10.4
	High	2.35
	Medium	0.577
	Low	0.153
	Very Low	0.037
	None	**0.001**
Prevail 22	Very High	9.61
	High	2.47
	Medium	0.589
	Low	0.147
	Very Low	0.035
	None	**0.001**
Prevail 25	Very High	10.8
	High	2.54
	Medium	0.647
	Low	0.169
	Very Low	0.037
	None	**0.001**
Prevail 26	Very High	10.3
	High	2.05
	Medium	0.551
	Low	0.147
	Very Low	0.036
	None	**0.002**

*Bold values are <LLOQ

Table 3.18: Spike and Recovery: Percent Recovery of Spiked Samples

Sample	Spike	% Recovery
Prevail 11	Very High	96
	High	84
	Medium	92
	Low	86
	Very Low	93
Prevail 12	Very High	99
	High	85
	Medium	88
	Low	86
	Very Low	100
Prevail 22	Very High	91
	High	89
	Medium	90
	Low	82
	Very Low	92
Prevail 25	Very High	102
	High	92
	Medium	99
	Low	95
	Very Low	98
Prevail 26	Very High	109
	High	93
	Medium	100
	Low	103
	Very Low	111

Table 3.19: Concentration of anti-EBOV GP Monomer Antibodies in IRF Samples*

Sample Name	Barcode	GP Monomer (EU/ml)
IRF 13	1-01374-3-02	42.2
IRF 14	1-00608-3-02	1289
IRF 15	1-01266-3-02	6967
IRF 16	1-01225-3-02	159
IRF 17	1-00325-3-02	10110
IRF 18	1-01520-3-02	2098
IRF 19	1-01597-3-02	1536
IRF 20	1-01616-3-02	590
IRF 21	1-01631-3-02	1036
IRF 22	1-01593-3-02	**13.7**
IRF 23	1-00275-3-02	380
IRF 24	1-02512-3-02	2056
IRF 25	1-01838-3-02	**10.3**
IRF 26	1-02087-3-02	**10.5**
IRF 27	1-02497-3-02	1348
IRF 28	1-02893-3-02	3569
IRF 29	1-02000-3-02	878
IRF 30	1-00295-3-02	**17.9**
IRF 31	1-02789-3-02	2093
IRF 32	1-00259-3-02	2387
IRF 33	1-02734-3-02	62.2
IRF 34	1-02810-3-02	25.6
IRF 35	1-03002-3-02	632
IRF 36	3-00158-4-02	703
IRF 37	3-00166-4-02	**12.2**
IRF 38	3-00325-4-02	7302
IRF 39	3-00342-4-02	2457
IRF 40	3-00366-4-02	233
IRF 41	3-00356-4-03	1430
IRF 45	3-00677-4-03	1996

*Bold values are <LLOQ

Table 3.20: Concentration of anti-EBOV GP Monomer Antibodies in IRF Samples*

Sample Name	Barcode	GP Monomer (EU/ml)
IRF 61	3955302	**11.6**
IRF 62	4258302	45.3
IRF 63	1891302	3086
IRF 64	1788302	10290
IRF 65	2078302	16560
IRF 66	2132302	34.7
IRF 67	2197302	86450
IRF 68	2236302	7764
IRF 69	2310302	5666
IRF 70	90070302	24130
IRF 71	2687302	**15.4**
IRF 72	2769302	271
IRF 73	3079302	6707
IRF 74	3085302	**12.1**
IRF 75	3263302	43.9
IRF 76	3561302	6123
IRF 77	3805302	2211
IRF 78	4296302	1413
IRF 79	128848301	1173
IRF 80	129521301	3224
IRF 81	129207301	2849
IRF 82	129830301	53.3
IRF 83	129742301	3705
IRF 85	129026301	32380
IRF 86	129046301	4099
IRF 87	128891301	10140
IRF 88	129380301	2360
IRF 91	129157301	1098
IRF 93	129258301	1315
IRF 95	129691301	1930

*Bold values are <LLOQ

REFERENCES

1. Rudge TL, Jr., Sankovich KA, Niemuth NA, Anderson MS, Badorrek CS, Skomrock ND, et al. Development, qualification, and validation of the Filovirus Animal Nonclinical Group anti-Ebola virus glycoprotein immunoglobulin G enzyme-linked immunosorbent assay for human serum samples. PLoS One. 2019;14(4):e0215457.
2. Prevac Study Team, Kieh M, Richert L, Beavogui AH, Grund B, Leigh B, et al. Randomized Trial of Vaccines for Zaire Ebola Virus Disease. N Engl J Med. 2022;387(26):2411-24.
